# Comparison of Zebrafish Embryo Toxicity and L929 Cytotoxicity Assays for Preliminary Quality-Control Assessment of Veterinary Autogenous Vaccine Matrices

**DOI:** 10.3390/vetsci13080786

**Published:** 2026-08-06

**Authors:** Antonella Di Paolo, Rosario Liberti, Lucia Anzalone, Claudia Colabella, Andrea Felici, Anita Emilia Colombo, Patrizia Bonfanti, Pamela Floris, Martina Pellegrini, Giulia Vita, Monica Cagiola

**Affiliations:** 1 Istituto Zooprofilattico Sperimentale dell’Umbria e delle Marche “Togo Rosati” Via Gaetano Salvemini 1, 06126 Perugia, Italy; r.liberti@izsum.it (R.L.); l.anzalone@izsum.it (L.A.); claudia.colabella@gmail.com (C.C.); a.felici@izsum.it (A.F.); m.pellegrini@izsum.it (M.P.); g.vita@izsum.it (G.V.); m.cagiola@izsum.it (M.C.); 2 Ospedale San Matteo degli Infermi, Usl Umbria 2, Via Loreto 3, 06049 Spoleto, Italy; 3 POLARIS Research Center, Department of Earth and Environmental Sciences, University of Milano-Bicocca, Piazza della Scienza 1, 20126 Milan, Italy; anita.colombo@unimib.it (A.E.C.); patrizia.bonfanti@unimib.it (P.B.); pamela.floris@unimib.it (P.F.)

**Keywords:** in vitro toxicity testing, cell viability, alternative methods, biological safety, QC batch release, bacterin vaccines

## Abstract

**Background**: Veterinary autogenous vaccines (AVs) are emergency tools produced in a short timeframe from bacterial/viral/fungal isolates when commercial vaccines are not available and undergo strict quality assurance and safety tests before release. In accordance with the European Directive 2010/63/EU and the 3R principles, we evaluated for the first time the possible substitution of the final toxicity in vivo test (abnormal toxicity test) with an alternative cell-based and embryo-based assay. **Methods**: The five most requested different autogenous matrices (adopted to produce a customizable number of lots requested from customers) were tested in vitro in parallel for residual toxicity by in vitro fish embryo acute toxicity (FET) test and cytotoxicity assay (MTS) using serial dilutions, together with the authorized in vivo test. **Results**: Statistical analysis revealed different outcomes between the two in vitro methods, highlighting the greater discriminatory power of the MTS assay compared to the other, with results more like the observed in vivo ATT outcome (pass/negative). **Conclusions**: In this preliminary first dataset, FET appeared poorly compatible with several complex autogenous vaccine matrices, while the L929 MTS assay yielded interpretable viability thresholds for all tested matrices. These findings support further qualification of MTS as part of an integrated non-animal batch characterization strategy, but they do not yet establish replacement of the in vivo test for veterinary autogenous vaccine quality control.

## Simple Summary

Batch safety testing of veterinary vaccines has historically relied on laboratory animals, although regulatory and ethical expectations increasingly require non-animal alternatives. This transition is possible thanks to different tests approved in recent decades and adopted as validated methods in Pharmacopeias. In this paper we explored the possible application of two in vitro assays for the residual toxicity of veterinary autogenous vaccines produced in Italy before release. Testing active parenteral products for residual toxicity is pivotal for quality-control assurance and end users’ safety. This study compared two possible non-animal approaches, an L929 cell-based MTS cytotoxicity assay and the zebrafish embryo FET test, using five bacterial autogenous vaccine matrices. The MTS assay appeared more compatible with these complex matrices, whereas FET showed high embryo mortality for several vaccines, possibly due to matrix-related physicochemical effects. Larger datasets and product-specific validation are required before implementation in batch-release quality control.

## 1. Introduction

Veterinary autogenous vaccines (AVs) are immunological products manufactured during emergency outbreaks when commercial alternatives are unavailable, as regulated by Italian Ministry Decree n. 287/1994 [1]. At the Istituto Zooprofilattico Sperimentale dell’ Umbria e delle Marche (IZSUM), customized bacterial and viral AVs are produced weekly and must undergo rigorous safety testing, encompassing assessments of general safety, residual pathogenicity, pyrogenicity, and cytotoxicity, before their release.

Historically, general batch safety has relied on the in vivo abnormal toxicity test (ATT), conducted in mice or guinea pigs [2]. However, this method relies on vague observational endpoints, yielding variable, non-specific, and non-reproducible data that are susceptible to false positives [3]. Consequently, regulatory frameworks are shifting toward alternative testing; for instance, the European Directorate for the Quality of Medicines & HealthCare (EDQM) has mandated the discontinuation of the in vivo Rabbit Pyrogen Test (RPT) by July 2025 [4]. Furthermore, international guidelines such as VICH GL59 advocate for a risk-based quality-control strategy to waive target animal safety testing when appropriate safety profiles are demonstrated [5].

In alignment with the 3Rs (Replacement, Reduction, and Refinement) and European Directive 2010/63/EU, the IZSUM is evaluating alternative non-animal safety tests [6]. The colorimetric L929 MTS assay offers a precise, quantifiable measure of cellular metabolic health, generating robust basal cytotoxicity data to potentially support in vivo testing waivers [7]. Concurrently, the zebrafish (Danio rerio) embryo model has gained prominence in toxicological research [8]. Zebrafish embryos develop rapidly, are optically transparent, and are not classified as protected experimental animals until 120 h post-fertilization (hpf), making the model ethically advantageous [9]. The OECD 236 fish embryo acute toxicity (FET) test utilizes these traits to evaluate lethality and sublethal toxicity, bridging the informational gap between in vitro results and complex in vivo mammalian testing [10].

Building upon previous evaluations of Gram-positive bacteria-based AVs [11], this study aims to assess the efficacy of the zebrafish FET test and the L929 MTS cytotoxicity assay as viable, non-mammalian alternatives for the preliminary toxicological screening of diverse veterinary AV matrices.

## 2. Materials and Methods

### 2.1. Veterinary Autogenous Vaccines

We selected the five most requested matrices among the authorized AVs produced at the Pharmaceutical Department in 2024 as previously described [12] for comparison (listed in Table 1, Supplementary Materials; compositions and patient information leaflets are presented in Supplementary Table S1). These matrices, which can be customized by end users in terms of batch size, packaging, and dosage according to specific veterinary prescriptions, covered five different target species (terrestrial and aquatic; ruminant, herbivore/omnivore, and carnivore) and two pathogen types (Gram-positive and Gram-negative).

Briefly, all vaccines were produced from bacterial strains isolated at the IZSUM Diagnostic Department from biological samples or following post mortem animal analysis. Single isolated colonies were then streaked onto blood agar or Tryptone Soy Agar (TSA) for identification via MALDI-TOF analysis and the antibiotic sensitivity test, and matrices were prepared using the appropriate culture media for each strain.

These culture media were produced in-house and their chemical compositions are reported in Supplementary Table S1; the final pH was 7.3 ± 0.2 for TSB and 7.4 ± 0.2 for the clostridia medium at 25 °C (Table S1).

For the Enterotoxemia AV, blood agar plates were used, followed by a clostridia-specific culture broth incubated under static anaerobic conditions for 48 h at 37 °C ± 2 °C.

For the Furunculosis AV, Tryptone Soy Agar (TSA) plates were adopted, followed by Tryptone Soy Broth (TSB) incubation with shaking for 24 h at 22 °C ± 2 °C.

For the Gangrenous Mastitis, Staphylococcosis and Streptococcosis AVs, TSA plates were also used followed by Tryptone Soy Broth incubation with shaking for 24 h at 37 °C ± 2 °C. All the culture media were produced at the Pharmaceutical Unit of the IZSUM.

After purity control, matrices were inactivated—with 0.04% (*v/v*) for 1 day for all except Clostridium perfringens, which received 0.05% formaldehyde (*v/v*) treatment for 7 days—under continuous stirring and then adjusted to their final concentrations according to the package leaflets as follows: 1.5 × 10^9^ CFU/mL for Gangrenous Mastitis and Staphylococcus, 1 × 10^9^ CFU/mL for Streptococcus, 9 × 10^8^ CFU/mL for Enterotoxemia, and 1 × 10^7^ CFU/mL for Furunculosis.

Each batch was assessed for free formaldehyde content (Spectrophotometric Quantification of Free Formaldehyde in Veterinary Vaccines [European Pharmacopoeia 10.0, Chapter 2.4.18, Method A]) before the toxicity assay, needing to comply with the threshold limit of 0.5 g/L.

### 2.2. Residual Toxicity Test (ATT)

The residual toxicity tests were performed in accordance with Italian Ministerial Decree (DM) 287/1994. For each autogenous vaccine (AV) batch, five mice weighing approximately 20 g each received a single 0.5 mL subcutaneous injection of the undiluted vaccine. Animal procedures were conducted under Ministerial Authorization PATVAX N. 7F31D.28 (Legislative Decree No. 26/2014, Supplementary Materials, original version in Italian All. VI—Pat Vax Art. 33), which limits the number of mice used annually to 2400.

To ensure objective welfare monitoring and guide timely interventions, a score-based clinical assessment framework was implemented for each vaccine batch, following the guidelines outlined in the European Commission’s “Working document on a severity assessment framework” (see Supplementary Table S2). Designated personnel monitored and inspected the animals daily for a one-week period (7 days).

### 2.3. Cytotoxicity Assay Design

#### 2.3.1. Cell Culture

The L929 cell line, derived from mouse connective tissue fibroblasts, was purchased from the Istituto Zooprofilattico Sperimentale della Lombardia e dell’Emilia Romagna (biobank code BS CL 56, passage 26). Cells were maintained in Minimum Essential Medium (MEM) (Euroclone, Pero, MI, Italy) supplemented with 10% fetal bovine serum (FBS, Gibco™, Grand Island, NY). Cultures were grown in 75 cm^2^ culture flasks and incubated at 37 °C ± 2 °C with 5% CO_2_, inspected daily, and then detached for the cytotoxicity assay using the TrypLE™ Express Enzyme (Gibco™) when 90% confluence was attained, as previously reported [11]. Finally, L929 cells were seeded at 15,000 cells/well in a 96-well microplate (Thermo Fisher Scientific, Waltham, MA, USA).

#### 2.3.2. MTS Assay

The experiments consisted of 5 independent sessions (one per matrix), each performed 7 times with each dilution in triplicate. To assess metabolic activity over 24 h, cells were exposed to each undiluted AV and to serial two-fold dilutions in culture media up to 1:2048 (two technical replicates per dilution for two conditions per AV). In each plate, a 1:10 aqueous sodium hypochlorite solution (16 replicates) served as a positive cytotoxicity control, whereas the negative control (NC) consisted of cells incubated in an FBS-only culture medium (32 replicates).

After the 24 h incubation, each well was inspected microscopically to qualitatively evaluate morphological signs of toxicity (Supplementary Figure S1) prior to quantitative analysis. Subsequently, the supernatants were replaced with the CellTiter 96^®^ AQueous One Solution Cell Proliferation Assay (MTS) reagent (Promega, Madison, WI, USA) and incubated for 3 h to allow soluble formazan product formation as an indicator of cell viability. Absorbance was measured spectrophotometrically at 492 nm using a Synergy HTX multimode reader (BioTek, Winooski, VT, USA) equipped with Magellan software version 6.01, followed by background subtraction using NC values. Viability was assessed after 24 h of contact, using the method previously described by Mosmann [7]. The vaccines’ effects on cell metabolic activity were expressed as percentages using the following formula: cell viability = (mean OD_492nm_ per dilution/NC OD_492nm_) × 100 (See Supplementary Graphs S1–S5 and Grids S1–S5 for dose–response curves and data.)

Finally, the first dilution that guaranteed at least a 70% cell viability response (critical dilution), after a specified exposure time, was established for each AV.

### 2.4. Fish Embryo Acute Toxicity Test

Adult AB wild-type zebrafish pairs from the European Zebrafish Resource Center (Karlsruhe Institute of Technology, Karlsruhe, Germany) were bred at the University of Milan-Bicocca facility (ethical approval ATS MetroMilano Prot. n. 0020984–12 Feburary 2018) using a ZebTec Active Blue recirculating system (Tecniplast, Buguggiate, Italy). Prior to the definitive FET assay, preliminary experiments were conducted to define the appropriate exposure conditions. Zebrafish embryos within 3 h post-fertilization (hpf) were exposed to undiluted AVs and their corresponding culture media.

Additional assays were performed to evaluate the contribution of formaldehyde to embryo toxicity, with embryos exposed to formaldehyde concentrations equivalent to those present in the 1.5% and 5% AV dilutions.

Moreover, 24 hpf embryos were manually dechorionated and exposed to a diluted AV formulation to assess the effect of chorion removal on exposure sensitivity. The information obtained from these preliminary experiments was used to establish the exposure conditions adopted in the definitive FET assay.

The FET tests were performed following OECD Guidelines No. 236 (2013), as described previously in Bonfanti et al. [13]. Briefly, in each plate, twenty wells were filled with 2 mL of a given AV (undiluted and diluted with fish water (in mg/L: NaHCO_3_ 100, Instant Ocean salt 100, CaSO_4_ 190). Negative (fish water) and positive control (dichloroaniline, DCA) plates were included in the experimental setup.

Embryos were collected within 3 h post-fertilization (hpf) and staged according to Kimmel et al. [14] [. Only fertilized embryos showing normal cleavage and embryonic development were used for subsequent exposure experiments.

One embryo was transferred to each well of the 24-well plate, with twenty embryos per plate exposed to the AV or culture medium at selected concentrations (from 1% to 7.5%) and four embryos maintained in fish water as internal negative controls. The embryos were subsequently maintained in a thermostatic chamber (Piardi, Italy) at 26 ± 0.5 °C under static conditions.

Every 24 h, embryos were monitored by observation under a stereo microscope for lethal endpoints (coagulation of fertilized eggs or lack of somite formation, detachment of the tail bud from the yolk sac, or heartbeat) according to endpoint-specific time points.

At 96 hpf (the test endpoint), data was collected for every sample, and the LC_50_ values (lethal concentration in 50% of exposed embryos) were extrapolated using probit analysis (IBM SPSS Statistics for Windows ver.27.0, Armonk, NY, USA: IBM Corp.).

All experiments were conducted in at least three independent experiments (*n* = 60 embryos per concentration total) and deemed valid only when control survival was ≥ 90% and positive-control mortality ≥ 30%; furthermore, as all procedures involved embryos within 120 hpf, they are not subject to animal experimentation regulation according to European Directive 2010/63/EU.

### 2.5. Statistical Analysis

For the MTS assay, cell viability was calculated for each independent experiment as the proportion of viable cells relative to the total number of cells seeded per well. For each vaccine matrix, values from the seven available independent experimental sessions were summarized using means and standard deviations, and 95% confidence intervals were constructed using the t distribution. The technical replicates within each experiment were averaged prior to analysis.

For the FET assay, embryo mortality was recorded at each concentration (from 1% up to a maximum of 7.5% dilution) and modeled using a probit regression with binomial error distribution for each matrix. Expected embryo survival was then derived as 1-*p*, applying the same linear transformation to the confidence-interval limits (delta method). The relationship between the two assays was evaluated descriptively by presenting viability (MTS) and mortality (FET) profiles for each matrix at the same concentrations.

All analyses were performed using Stata 16.1 (StataCorp, College Station, TX, USA).

## 3. Results

### 3.1. Residual Toxicity Test (ATT)

All matrices tested in mice passed the in vivo assay, with no general or local adverse reactions recorded and clinical scores under 6 (Supplementary Table S2).

### 3.2. MTS Assay

Among the AV concentrations tested, the Gangrenous Mastitis and Staphylococcus vaccines ensured at least 70% of cells were viable at a concentration of 1.56% (1:64 dilution), Furunculosis and Streptococcus at 0.78% (1:128 dilution), and Enterotoxemia at 6.25% (corresponding to a 1:16 dilution), as listed in Table 2 and Supplementary Graphs S1–S5.

### 3.3. Fish Embryo Test

Exposing embryos to undiluted AVs and their corresponding culture media resulted in 100% mortality within 24 hpf (unpublished data ). To assess whether residual formaldehyde contributed to toxicity, embryos were exposed to formaldehyde concentrations equivalent to those present in 1.5% and 5% AV dilutions; no mortality was observed. In contrast, TSB alone induced 20% mortality at 1.5% dilution and 100% mortality at 5%, indicating intrinsic medium toxicity. Furthermore, exposing 24 hpf dechorionated embryos to a 1.5% dilution of furunculosis AV resulted in 48% mortality, comparable to that observed in chorion-intact embryos (52%), suggesting that AV-induced toxicity is not mediated by chorion interactions affecting gas exchange.

The FET test results are reported in Supplementary Figure S2, while the AVs 96 hpf-LC50, -LC30 and -LC10 values are reported in Table 3.

For the bovine Enterotoxemia AV, no significant increase in mortality was observed at the lowest concentrations compared with the negative control. However, mortality rose sharply at 5% dilution, reaching 63%, and increased further to 100% at the highest concentration tested (7.5%). Mortality induced by the corresponding culture medium at the same dilution was higher than by the AV.

The Furunculosis AV caused 52% and 100% mortality at 1.5% and 5% dilutions, respectively. While the corresponding TSB medium showed a comparable dose-dependent toxic effect, the significantly higher mortality at 1.5% and 2.5% in vaccine-exposed embryos suggests an additional toxic contribution from vaccine components.

Dose-dependent toxicity was also recorded for the Bovine Enterotoxiemia and Gangrenous Mastitis vaccine, with mortality reaching 40% and 100% at 1.5% and 7.5% dilutions. The overall toxicity profile paralleled that of the corresponding culture medium, even if mortality at 1.5% was significantly lower in the vaccine-treated group than in embryos exposed to the medium alone.

Finally, the rabbit Staphylococcosis and swine Streptococcosis vaccines showed significant mortality values at 1% concentration versus negative controls, reaching 100% at 5% dilution. For rabbit Staphylococcosis, mortality generally followed the pattern observed for the corresponding culture medium; however, for swine Streptococcus, significantly higher mortality was detected at 1% and 2.5% in comparison with the medium alone at the same dilutions.

### 3.4. Statistical Analysis

MTS and FET survival results were evaluated considering the same vaccine concentration, established to figure out a non-effective and safe concentration that guarantees the 70% cell viability for MTS (Table 4).

As shown in Table 4, for the MTS method, only the Enterotoxemia AV reached 70% vitality at 6.25%; the other vaccines reached this threshold at lower concentration percentages, specifically Furunculosis and Streptococcosis at 0.78% and Gangrenous Mastitis and Staphylococcosis at 1.56%.

In parallel, considering FET at the same AV concentrations, we observed that three out of five matrices exceeded the 70% survival viability, while only two were under the threshold.

## 4. Discussion and Conclusions

### 4.1. The Shift Toward Alternative Safety Testing Models

The traditional abnormal toxicity test (ATT) required for AV quality assurance often presents a disconnect between the effects observed in laboratory animals and the potential adverse effects in the target species [3]. The ATT is highly variable, resource-consuming, and ethically limited, frequently leading to false positives and unnecessary discards of promising batches [3]. In accordance with the 3Rs, alternative models should replace animal-based studies whenever they provide equivalent toxicological information [6,15]. Consequently, cell-based models like the L929 MTS assay (ISO 10993) and the zebrafish FET test (OECD 236) have been developed to reduce reliance on live animal testing [7,10,16].

The zebrafish embryo model is particularly valuable as it bridges the gap between basic in vitro cell assays and whole-organism responses, offering physiological similarities to humans, innate and adaptive immune systems, and high-throughput capabilities without being subjected to protected animal regulations prior to 120 hpf [9,17].

### 4.2. Comparative Assessment of MTS and FET Assays

Despite their common regulatory aim, the MTS and FET assays produced markedly different outcomes when evaluating complex AV matrices. Overall, direct exposure of cultured L929 cells to AV dilutions resulted in higher viability at lower concentrations compared to the zebrafish embryo model. The MTS assay results closely mirrored the realistic outcomes of the in vivo ATT, in which none of the analyzed vaccine matrices caused general or local adverse reactions.

Conversely, the zebrafish embryos demonstrated high mortality rates, and a comparison of survival indices revealed that only the Gangrenous Mastitis AV yielded similar results between the two methods (0.773 for MTS and 0.764 for FET). The greatest discrepancy was observed in the Enterotoxemia AV, which showed an MTS survival index of 0.782 versus a FET index of 0.126. Interestingly, the autogenous vaccines generally elicited higher toxicity than their respective culture media alone in the FET assay, suggesting that culture medium cannot solely explain the observed embryo mortality. Preliminary investigations confirmed that residual formaldehyde, at the concentrations present in the ready-to-use vaccines, was not toxic to the embryos. Furthermore, experiments utilizing dechorionated embryos demonstrated that the vaccine components do not induce toxicity by interfering with the chorion or its gas-exchange functions.

### 4.3. Matrix Complexities in the Zebrafish Model

These findings suggest that zebrafish embryos are not the most suitable model for evaluating the toxicity of these highly complex autogenous vaccines. The elevated organic load of the AV preparations may disrupt the osmotic equilibrium between the water and biological components or reduce dissolved oxygen levels, thereby compromising embryo survival independently of any intrinsic vaccine-related toxicity. Additionally, while zebrafish larvae offer high-throughput screening potential, they may not accurately represent adult toxicities [3]. Adult zebrafish could theoretically be used to evaluate general organ safety to meet global regulatory requirements, though this approach would sacrifice testing throughput [3,17].

### 4.4. A Tiered Quality-Control Strategy

The harmonization guidelines established by VICH GL59 strongly advocate for setting criteria to waive Laboratory Animal Batch Safety Testing (LABST) [5].

Applying these waivers to veterinary AVs presents a significant regulatory challenge, as these rapid-response formulations lack the long-term statistical consistency and standardized batch histories typical of commercial vaccines. To navigate this hurdle, the L929 MTS assay provides exceptional value as an accessible, high-throughput platform to quantify basal cytotoxicity. It serves as a sensitive initial step in a risk-based testing paradigm to evaluate residual inactivating agents and adjuvants, flagging problematic batches early in the production cycle without utilizing mammals.

### 4.5. Conclusions

The transition away from animal-based assays for AV safety assurance relies on accumulating data from multiple integrated in vitro methodologies [18]. Combining cell-based viability assays with evaluations of free formaldehyde content and bacterial endotoxin loads [19] can provide comprehensive batch characterization prior to release. While the zebrafish FET assay demonstrated limitations when faced with complex matrices, the L929 MTS assay yielded interpretable and highly relevant viability thresholds. However, further product-specific validations and analyses are required to define valid cut-offs for individual vaccine lots. Ultimately, integrating these robust in vitro methods alongside established Correlates of Protection will successfully define the safety profile of customized AVs while strictly adhering to the 3R principles [6,15].

## Figures and Tables

**Table 1:** Veterinary autogenous vaccine matrices evaluated in this study.

Avs	Pathogen	Target Species
Enterotoxemia	*Clostridium perfringens type A*	Bovine
Furunculosis	*Aeromonas salmonicida salmonicida*	Salmonid
Gangrenous mastitis	*Staphylococcus aureus*	Ovine
Staphylococcosis	*Staphylococcus aureus*	Rabbit
Streptococcosis	*Streptococcus suis*	Swine

**Table 2:** Critical concentration (≥70% cell viability), mean, and standard deviation (SD) of MTS-derived cell viability for each vaccine matrix. Each matrix was tested individually in 7 independent experiments (2 technical replicates each) at a seeding density of 15,000 cells/well.

AVs	AV Concentration %	Mean	SD
Enterotoxemia	6.25	0.782	0.084
Furunculosis	0.78	0.861	0.096
Gangrenous mastitis	1.56	0.773	0.051
Staphylococcosis	1.56	0.788	0.058
Streptococcosis	0.78	0.855	0.120

**Table 3:** Lethal concentration at 96 h post-fertilization (96 hpf- LC_50_, LC_30_ and LC_10_) after exposure of zebrafish embryos to AVs. The 96 hpf-LC values are expressed as dilution percentages of the sample. * denotes a value that could not be calculated.

Avs	96 hpf-LC_50_ (CI 95%)	96 hpf-LC_30_ (CI 95%)	96 hpf-LC_10_ (CI 95%)
Enterotoxemia	4.26 (3.90–4.66)	3.35 (2.99–3.70)	2.03 (1.57–2.42)
Furunculosis	1.82 *	1.46 *	*
Gangrenous mastitis	2.94 (1.14–7.45)	1.93 (0–4.15)	0.48 (0–2.06)
Staphylococcosis	1.55 (1.43–1.67)	1.16 (1.04–1.28)	0.61(0.44–0.76)
Streptococcosis	1.49 (*)	1.00 (*)	0.28 (*)

**Table 4:** Results of MTS survival and FET-predicted survival at MTS critical concentrations (mean and 95% confidence interval).

Avs	Concentration (%)	MTS Survival (CI 95%)	FET Survival (CI 95%)
Enterotoxemia	6.25	0.782 (0.704–0.860)	0.126 (0.058–0.194)
Furunculosis	0.78	0.861 (0.772–0.950)	0.938 (0.896–0.980)
Gangrenous mastitis	1.56	0.773 (0.726–0.820)	0.764 (0.721–0.807)
Staphylococcosis	1.56	0.788 (0.735–0.842)	0.493 (0.425–0.560)
Streptococcosis	0.78	0.853 (0.744–0.966)	0.775 (0.723–0.826)

## Data Availability

The data presented in this study are available on request from the corresponding author.

## Supplementary Materials

The following supporting information can be downloaded at https://www.mdpi.com/article/10.3390/vetsci13080786/s1, Supplementary Table S1: Chemical composition of the culture media produced at the Pharmaceutical Department and used in the manufacture of the vaccine types involved in the trial; Supplementary Table S2: Clinical Scoring Table for Animal Welfare Monitoring; Supplementary Graph S1: Bovine Enterotoxemia; Supplementary Graph S2: Furunculosis; Supplementary Graph S3: Gangrenous Mastitis; Supplementary Graph S4: Staphylococcosis; Supplementary Graph S5: Streptococcosis; Supplementary Grid S1: Bovine Enterotoxemia Cell viability (Vitality) = (mean OD_4__9__2__n__m_ per dilution/NC OD_4__9__2__n__m_) × 100; Supplementary Grid S2: Furunculosis Cell viability (Vitality) = (mean OD_4__9__2__n__m_ per dilution/NC OD_4__9__2__n__m_) × 100; Supplementary Grid S3: Gangrenous Mastitis Cell viability (Vitality) = (mean OD_4__9__2__n__m_ per dilution/NC OD_4__9__2__n__m_) × 100; Supplementary Grid S4: Staphylococcosis Cell viability (Vitality) = (mean OD_4__9__2__n__m_ per dilution/NC OD_4__9__2__n__m_) × 100; Supplementary Grid S5: Cell viability (Vitality) = (mean OD_4__9__2__n__m_ per dilution/NC OD_4__9__2__n__m_) × 100; Supplementary Figure S1: Criteria of toxicity effect based on changes in cell morphology [20]; Supplementary Figure S2: Embryotoxicity of AVs and their corresponding media.
