# Supplementary material for: Comparison of Zebrafish Embryo Toxicity and L929 Cytotoxicity Assays for Preliminary Quality-Control Assessment of Veterinary Autogenous Vaccine Matrices
Source: Vet Sci. 2026 Aug 6;13(8):786. doi: 10.3390/vetsci13080786 (PMC13517733; doi:10.3390/vetsci13080786)

Supplementary Table S1. Chemical composition of the culture media produced at the Pharmaceutical Department and used in the manufacture of the vaccine types involved in the trial.

Component	Vaccine TSB	Clostridia Medium
Deionized water	1000 mL	1000 mL
Tryptone Soya Broth	30 g	—
Vegetable peptone	—	20 g
Disodium phosphate dihydrate	—	3.13 g
Sodium chloride	—	5.0 g
Yeast extract	—	1.0 g
L-(+)-cysteine	—	2.0 g
Glucose	—	5.0 g

Supplementary Table S2
Clinical Scoring Table for Animal Welfare Monitoring

Macro-category	Area of Observation	Specific Clinical Indicator	Score
**Appearance**	Coat condition	Slightly ruffled coat	1
		Mild piloerection	2
		Marked piloerection	3
**Body functions**	Respiration	Accelerated (tachypnea)	1
		Labored (hyperpnea)	2
		Severely labored (dyspnea)	3
**Environment**	Feces	Soft stools	1
		Diarrhea	2
		Bloody diarrhea	**HEP**
**Behavior**	Undesirable behaviors	Repetitive behavior (stereotypies)	1
		Hair and whisker trimming (*barbering*)	2
		Increased aggression	3
**Behavior**	Posture and motility	Abnormal posture	1
		Abnormal gait, lameness, lethargy	2
		Uncoordinated movements and reluctance to move	3
		Abdominal retraction and tilted head with absence of movement	**HEP**

Macro-category	Area of Observation	Specific Clinical Indicator	Score
**Procedure-specific**	Injection site	Slightly reactive (localized inflammation)	1
		Markedly reactive (inflammation and tissue edema)	2
		Severely hyperergic with alopecia and skin erythema (redness)	3

Note: cumulative scores ≥6 trigger humane endpoints.

The scoring system evaluated four primary macro-categories: Appearance: Coat and skin condition, Body functions: Respiratory rate and quality; Environment: Fecal consistency and cage conditions; Behavior: Posture, motility, and the manifestation of anomalous behaviors.

Additionally, the injection site was visually inspected daily as a procedure-specific indicator for local reactions. Any mild-to-moderate clinical signs attributable to pain or distress were immediately recorded and managed according to established humanitarian endpoints. Final AV quality assurance and batch validation were determined by the complete absence of acute, local, or systemic adverse reactions; the occurrence of any such reactions constituted the batch failure fail criteria.

Supplementary Graphs. Dose response curve for Avs analysed.

Graph S1 Bovine Enterotoxemia

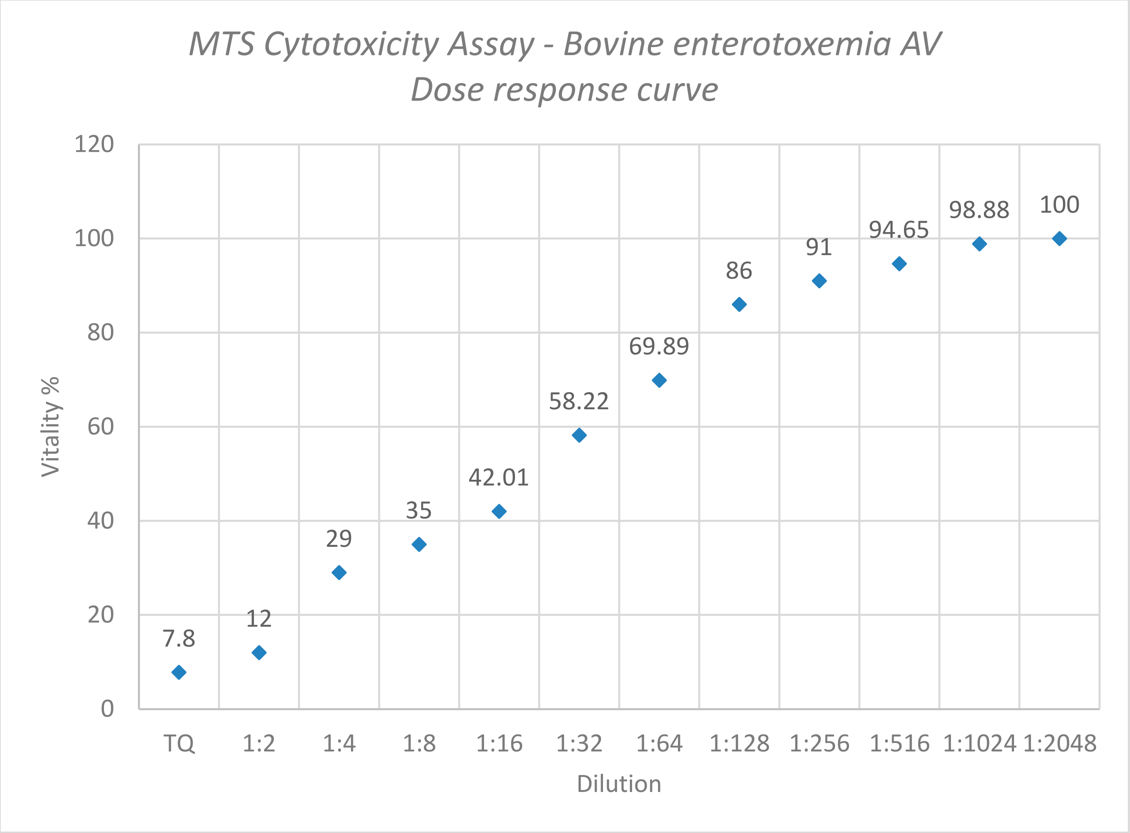

Grid S1 Bovine Enterotoxemia Cell viability (Vitality)= (mean OD_{492nm} per dilution / NC OD_{492nm}) × 100

**AVs dilution(n1-n13)**	**OD492nm mean value**	**% Vitality**
Undiluted	0.2	0.85
1:2	0.3	12.87
1:4	0.7	30
1:8	0.9	38.62
1:16	1.0	42.91
1:32	1.37	58.79
1:64	1.61	69
1:128	2.01	86
1:256	2.14	91
1:516	2.2	94.65
1:1024	2.30	98.88
1:2048	2.33	100
NC (cell untreated)	2.33	100
PC (cell treated)	0.04	0.01

Graph S2 Furunculosis

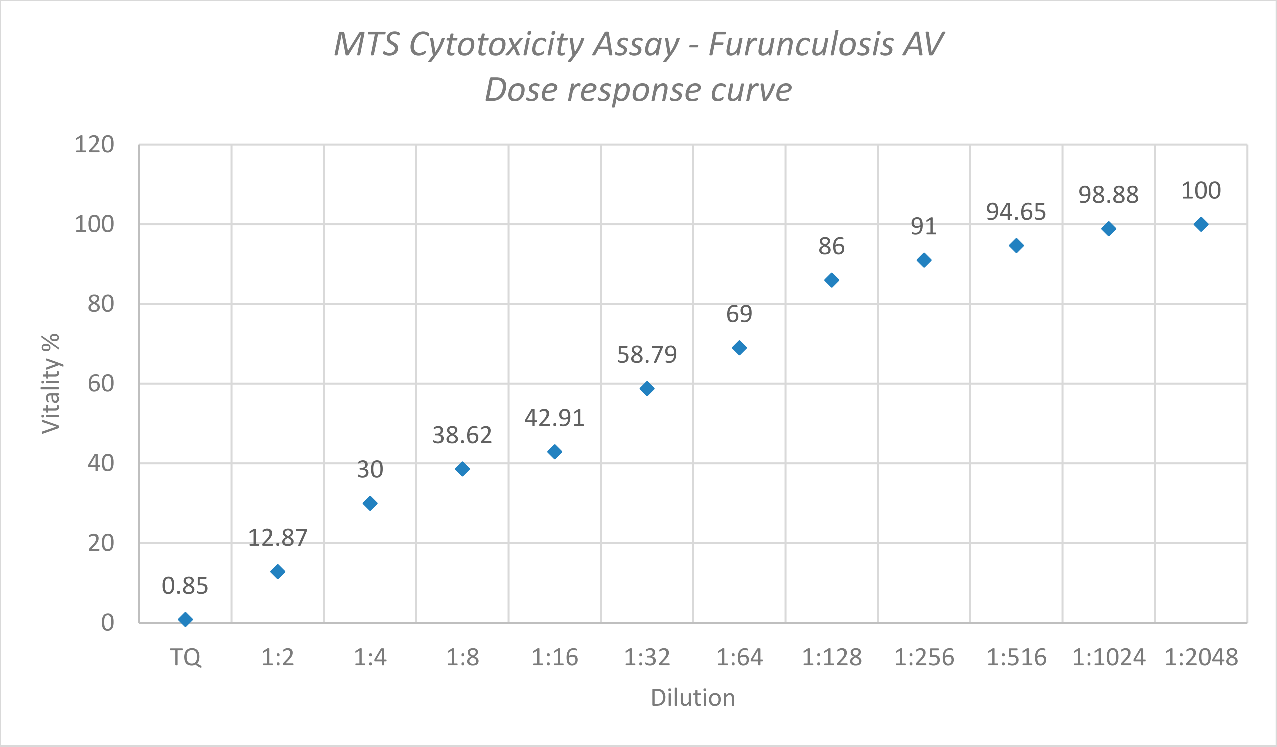

Grid S2 Furunculosis Cell viability (Vitality)= (mean OD_{492nm} per dilution / NC OD_{492nm}) × 100

**AVs dilution(n1-n13)**	**OD492nm mean value**	**% Vitality**
Undiluted	0.2	0.85
1:2	0.3	12.87
1:4	0.7	30
1:8	0.9	38.62
1:16	1.0	42.91
1:32	1.37	58.79
1:64	1.61	69
1:128	2.01	86
1:256	2.14	91
1:516	2.2	94.65
1:1024	2.30	98.88
1:2048	2.33	100
NC (cell untreated)	2.33	100
PC (cell treated)	0.04	0.01

Graph S3 Gangrenous Mastitis

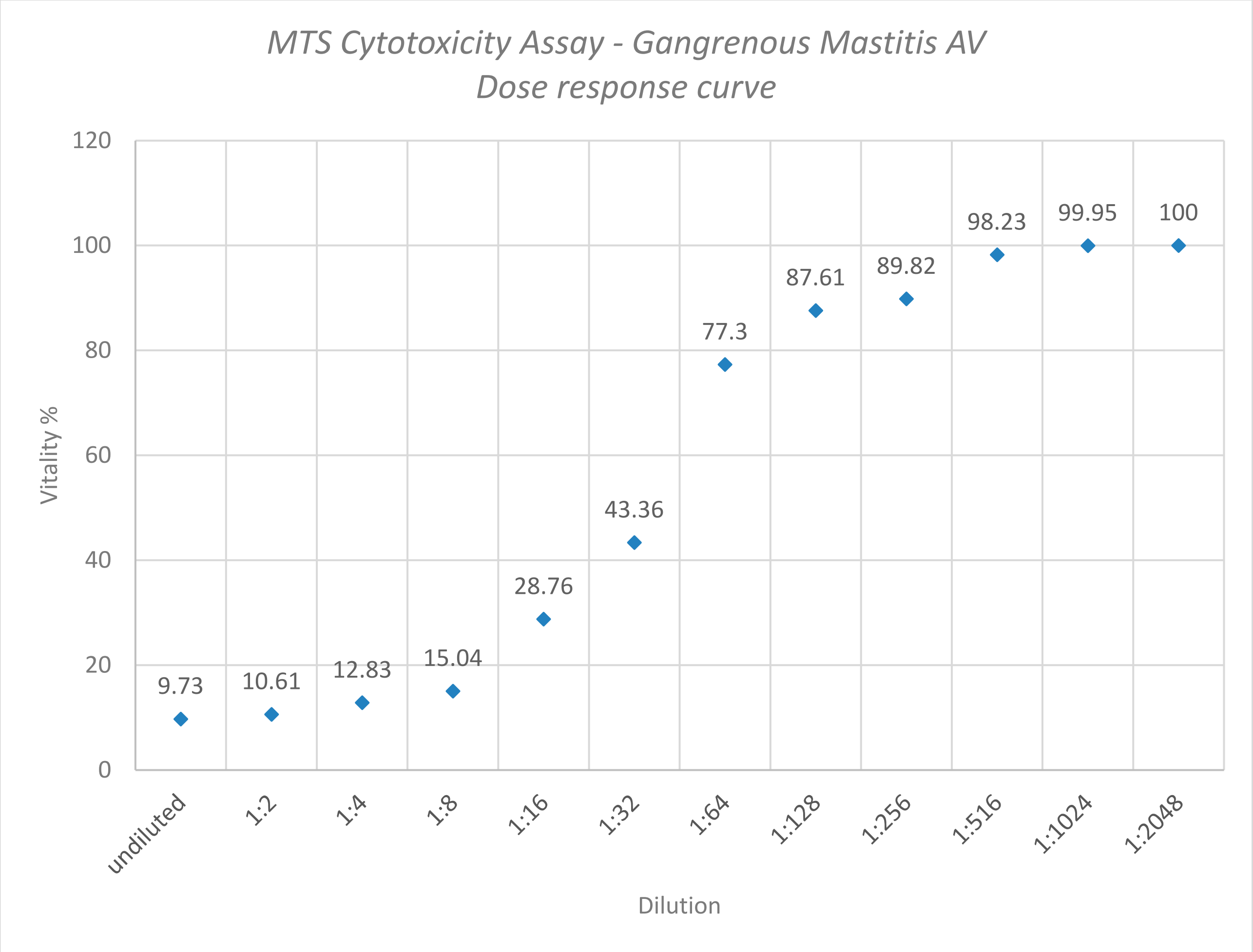

Grid S3 Gangrenous Mastitis Cell viability (Vitality)= (mean OD_{492nm} per dilution / NC OD_{492nm}) × 100

AVs dilution(n1-n13)	OD492nm mean value	% Vitality
Undiluted	0.22	9.73
1:2	0.24	10.61
1:4	0.29	12.83
1:8	0.34	15.04
1:16	0.65	28.76
1:32	0.98	43.36
1:64	1.75	77.3
1:128	1.98	87.61
1:256	2.03	89.82
1:516	2.22	98.23
1:1024	2.25	99.95
1:2048	2.26	100
NC (cell untreated)	2.26	2.26
PC (cell treated)	0.05	2.21

Graph S4 Staphylococcosis

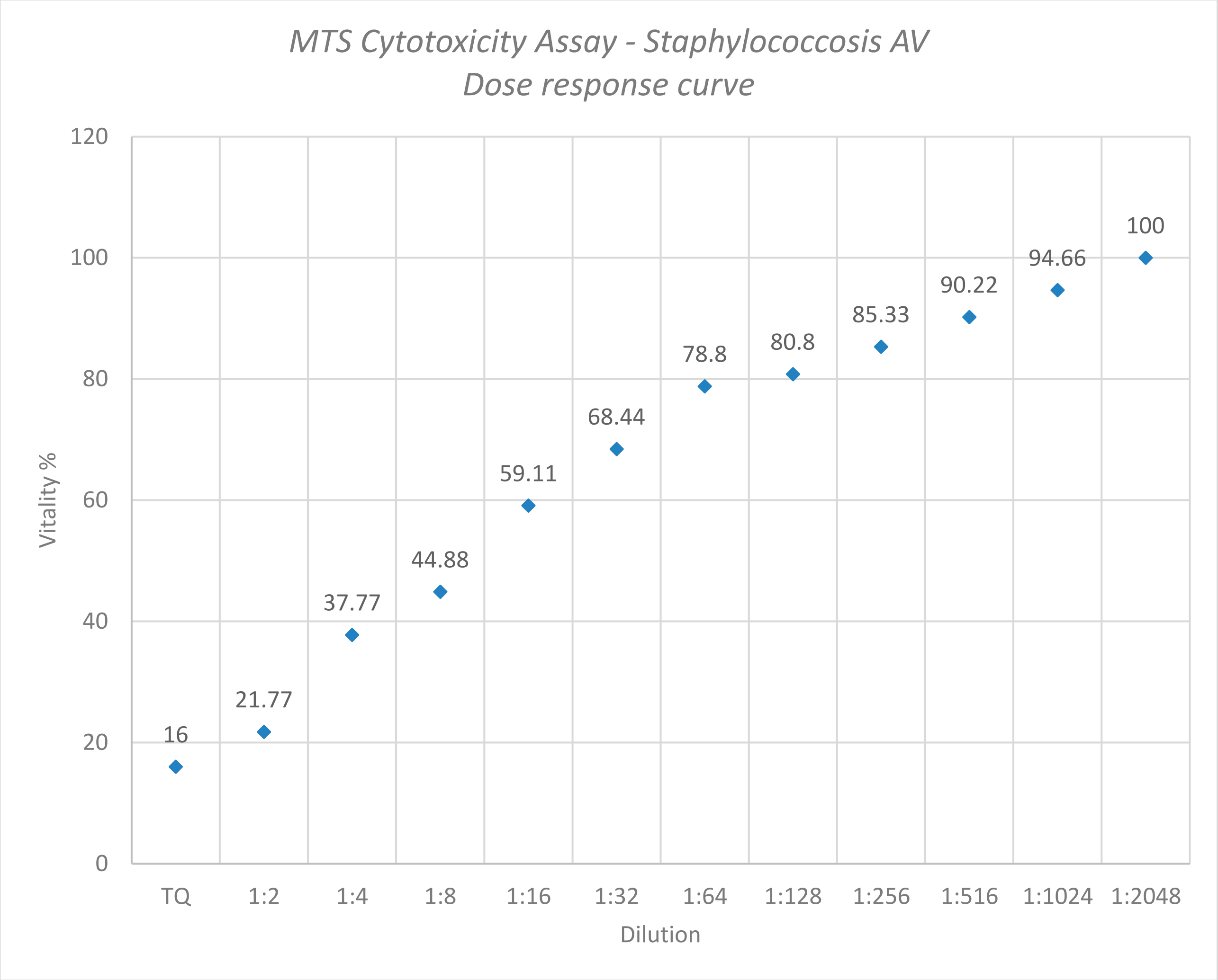

Grid S4 Staphylococcosis Cell viability (Vitality)= (mean OD_{492nm} per dilution / NC OD_{492nm}) × 100

**AVs dilution(n1-n13)**	**OD492nm mean value**	**% Vitality**
Undiluted	0.36	16
1:2	0.49	21.77
1:4	0.85	37.77
1:8	1.01	44.88
1:16	1.33	59.11
1:32	1.54	68.44
1:64	1.77	78.8
1:128	1.82	80.88
1:256	1.92	85.33
1:516	2.03	90.22
1:1024	2.13	94.66
1:2048	2.25	100
NC (cell untreated)	2.25	2.26
PC (cell treated)	0.08	2.21

Graph S5 Streptococcosis

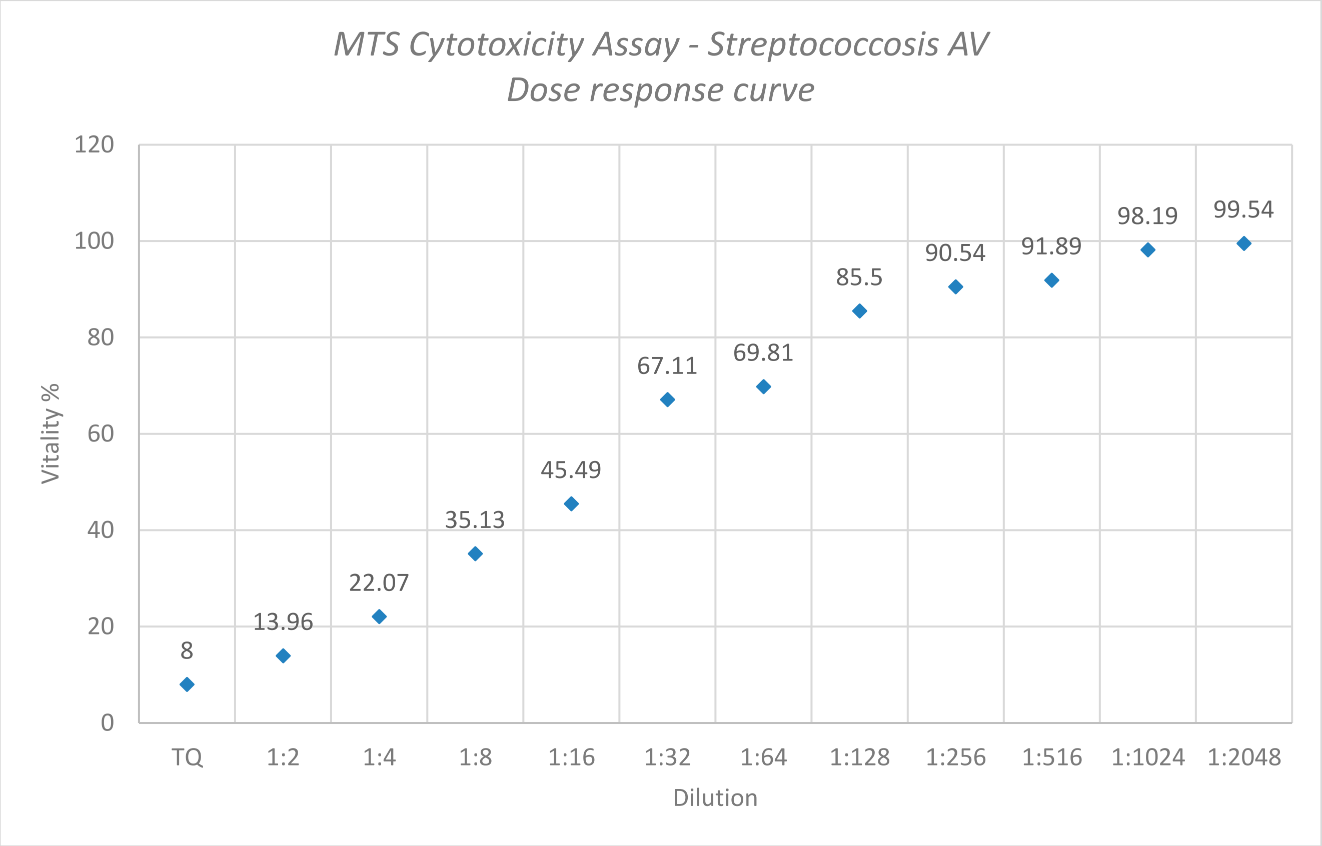

Grid S5 Cell viability (Vitality)= (mean OD_{492nm} per dilution / NC OD_{492nm}) × 100

**AVs dilution(n1-n13)**	**OD492nm mean value**	**% Vitality**
Undiluted	0.19	8
1:2	0.31	13.96
1:4	0.49	22.07
1:8	0.78	35.13
1:16	1.01	45.49
1:32	1.49	67.11
1:64	1.55	69.81
1:128	1.9	85.5
1:256	2.01	90.54
1:516	2.04	91.89
1:1024	2.18	98.19
1:2048	2.21	99.54
NC (cell untreated)	2.22	100
PC (cell treated)	0.04	0.06

Supplementary Figure. S1 Criteria of toxicity effect based on changes in cell morphology.

Table 4. Criteria of toxicity effect based on changes in cell morphology [12].

Grade	Toxicity	Cell Morphology
0	lack	Discrete intracytoplasmic granules, no evidence of cell lysis, lack of inhibition of cell growth
1	weak	No more than 20% of rounded, shrunk cells, separating from the substrate without densities of cytoplasm, individual cells disrupted
2	moderate	No more than 50% of rounded cells, no evidence of granules, vast cell lysis and empty spaces between cells
3	average	No more than 70% of rounded cells, cells underwent lysis
4	strong	Almost completely or completely damaged cell culture

Reference: Szymonowicz, M., Korczynski, M., Dobrzynski, M., Zawisza, K., Mikulewicz, M., Karuga-Kuzniewska, E., ... & Wiglusz, R. J. (2017). Cytotoxicity evaluation of high-temperature annealed nanohydroxyapatite in contact with fibroblast cells. *Materials*, *10*(6), 590.

Supplementary Figure. S2. Embryotoxicity of AVs and their corresponding media. *p<0.05 and **p<0.01 vs. control, ##p<0.01 vs. the same dilution of the corresponding vaccine or culture medium (ANOVA + Fisher LSD Method).

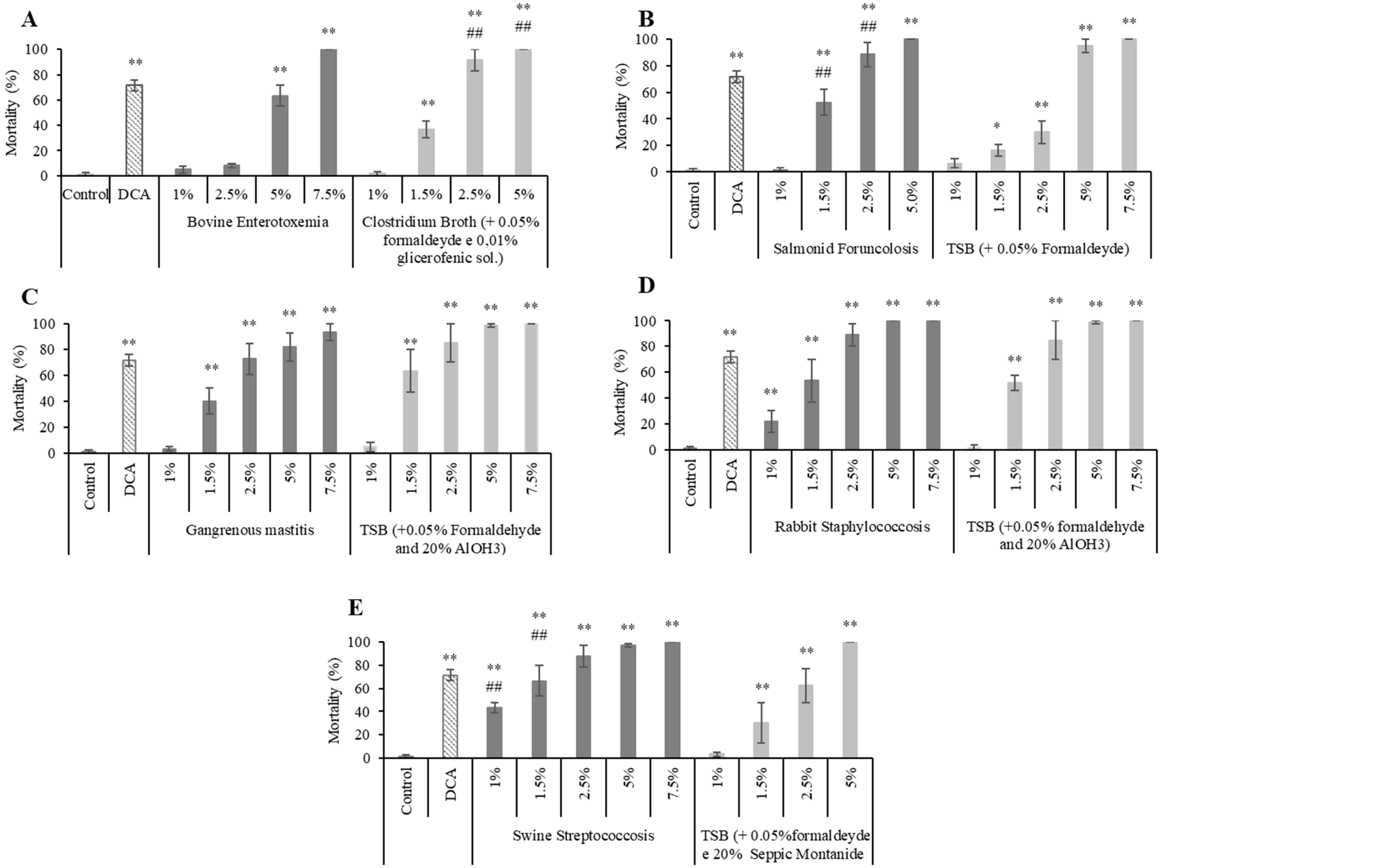

Supplement: Supplementary file 1 [file vetsci-13-00786-s001.zip › vetsci-4400218-supplementary.pdf]
